# Aromatic amine exposure and bladder cancer: a review of recent advances in epidemiology, pathogenesis, and prevention

**DOI:** 10.3389/fcell.2026.1914792

**Published:** 2026-09-22

**Authors:** Shuxin Li, Yueqiu Zhang, Tie Mao, Jiaqi Wei, Wenyao Zhang, Jingqi Zhang, Wei Wei

**Affiliations:** 1 Department of Urology, First Hospital of Jilin University, Changchun, China; 2 Department of Otorhinolaryngology, Head and Neck Surgery, West China Hospital, Sichuan University, Chengdu, China; 3 Operating Room 1, First Hospital of Jilin University, Changchun, China; 4 Department of Hepatobiliary and Pancreatic Surgery I, First Hospital of Jilin University, Changchun, China; 5 Department of Urology, Shengjing Hospital, China Medical University, Shenyang, China

**Keywords:** aromatic amines, bladder cancer, DNA adducts, genetic susceptibility, metabolic activation, risk prediction

## Abstract

Aromatic amines are environmental and occupational pollutants found in industrial chemicals, tobacco smoke, hair dyes, and azo-dye-containing textiles. Epidemiological studies confirm that occupational settings and tobacco smoking are the primary sources of exposure, with inhalation and dermal absorption being the major routes of entry, significantly increasing bladder cancer risk. Mechanistically, aromatic amines undergo metabolic activation, leading to DNA adduct formation, oxidative stress, DNA double-strand breaks, and activation of oncogenic signaling pathways such as MAPK/AP-1. Genetic polymorphisms in metabolic enzymes modify individual susceptibility, and risk prediction models incorporating these markers show promise. However, controversies remain regarding low-dose combined exposures, differences in carcinogenic potency among aromatic amines, and inadequate control for confounders in epidemiological studies. Future research should use multi-omics technologies to elucidate complete molecular networks and establish integrated prevention strategies.

## 1 Introduction

Bladder cancer is a common malignancy worldwide, and exposure to aromatic amines has been firmly established as a major environmental and an occupational risk factor (Lobo et al., 2022; Filho et al., 2026). Bladder cancer ranks ninth or 10th among all malignant tumors by incidence, depending on the statistical criteria used (Lobo et al., 2022; Filho et al., 2026). In 2022, the number of new cases of bladder cancer worldwide reached approximately 613,791 (Zhao et al., 2024). In terms of geographic and gender distribution, the incidence of this disease is relatively higher in the United States, where it ranks sixth among all cancers (Flaig et al., 2026; Flaig et al., 2024). In Asia, bladder cancer ranks eighth among malignant tumors in men (Zhao et al., 2024). There are gender differences in the prevalence of bladder cancer, with a higher incidence among men (Chaudhary et al., 2025). Urothelial carcinoma is the predominant histological type, accounting for over 90% of cases, and exhibits significant histological heterogeneity (Flaig et al., 2024; Chaudhary et al., 2025). Aromatic amines are polar organic compounds consisting of an amino group linked directly to an aromatic ring; they are widely present in industrial chemicals, tobacco smoke, hair dyes, and azo-dye-containing textiles (Alouini, 2024; Tajima et al., 2020; Nakano et al., 2025). Certain members have low molecular weight and high reactivity, making them readily metabolized into electrophilic intermediates that form DNA adducts. The International Agency for Research on Cancer (IARC) has classified 2-naphthylamine and 4-aminobiphenyl as human bladder carcinogens (Alouini, 2024; Tajima et al., 2020). Human exposure originates mainly from occupational settings and tobacco smoke, with inhalation and dermal contact serving as the primary routes of uptake, with additional contributions from diet, hair dyes, and textile washing (Nakano et al., 2025; Souza et al., 2023; Hosseini et al., 2023). Endogenous sources of aromatic amines primarily depend on the metabolic activity of the gut microbiota, among which several genera within the phylum Firmicutes can produce aromatic amines through decarboxylation of aromatic amino acids, thereby serving as a major source of aromatic amines in the colonic lumen (Sugiyama et al., 2022). Furthermore, during human metabolism, cytochrome P450 enzymes can convert certain precursors into active aromatic amine derivatives; these metabolites may reach the bladder via the circulatory system and subsequently contribute to carcinogenesis (Zhang et al., 2022). Although substantial epidemiological evidence links aromatic amines to bladder cancer, the complete molecular mechanisms, population-attributable risks of different exposure routes, and the biological basis for gender and geographical differences remain to be fully elucidated. This mini review summarizes recent advances in metabolic activation, DNA damage, signaling pathways, genetic susceptibility, risk prediction, and future intervention strategies.

## 2 The epidemiological link between aromatic amines and bladder cancer

Both occupational settings and non-occupational sources, such as tobacco smoke, contribute significantly to human exposure. This section summarizes the key epidemiological findings linking aromatic amines to bladder cancer risk.

### 2.1 Evidence linking occupational sources to the risk of bladder cancer

Epidemiological studies consistently identify occupational sources of aromatic amines as a major risk factor for bladder cancer. Workers in industries such as dye, rubber, petrochemicals, and metal processing face significantly elevated risks. For example, an epidemiological study in Japan of workers exposed to aromatic amines in dye factories showed that the standardized incidence ratio (SIR) for bladder cancer among those exposed to o-toluidine reached 56.8, with a clear dose–response relationship; although the SIR for other aromatic amines was also elevated, the dose–response relationship was unclear (Nakano et al., 2021). The Iranian case-control study (involving 717 bladder cancer patients and 3,477 controls) found that male metalworkers and workers potentially exposed to aromatic amines had a significantly increased risk of bladder cancer. In contrast, administrative staff and clerical workers had a reduced risk (Hosseini et al., 2023). A prospective cohort study from the Agricultural Health Study further supported this association, showing that imidazolinone-based aromatic amine herbicides significantly increased bladder cancer risk, particularly among non-smokers (Koutros et al., 2016). Cross-sectional studies in Lebanon and Jordan also reported high occupational exposure to aromatic amines, further supporting the universality of this association across populations (Jabra et al., 2021). Likewise, a cohort study of 1,875 workers at a rubber chemical manufacturing plant identified o-toluidine as the primary suspected carcinogen. Additionally, Japanese workers developed bladder cancer 10–40 years after dermal exposure to the IARC Group 1 carcinogen 4,4′-methylenebis (Kobayashi et al., 2025). A 73-year Italian cohort follow-up study involving 590 workers exposed to aromatic amines in dye factories showed a standardized mortality ratio (SMR) for bladder cancer of 14.86, indicating a mortality rate nearly 15 times higher than that of the general population (Ciocan et al., 2022). The risk increased with younger age at first exposure and longer exposure duration. Furthermore, the SMR remained as high as 3.5 even 30 years after exposure ceased, indicating that the risk persists over the long term (Ciocan et al., 2022). *In vitro* studies demonstrate that various aromatic amines, including o-toluidine, can rapidly penetrate human skin and accumulate in the bladder after excretion, thereby increasing cancer risk (Rozanec and Secin, 2020; Konorev et al., 2023).

### 2.2 Tobacco smoke as a major non-occupational source of aromatic amines and bladder cancer risk

Tobacco smoke is a major source of non-occupational exposure to aromatic amines. It contains several known or suspected bladder carcinogens, including 4-aminobiphenyl, 2-naphthylamine, and o-toluidine (Konorev et al., 2023; Mazumder et al., 2019; Mazumder et al., 2023). Smokers excrete significantly higher urinary levels of these compounds than non-smokers, with levels positively correlated with daily cigarette consumption (Mazumder et al., 2019; Scherer et al., 2022). A randomized controlled trial confirmed that cigarette smokers have markedly higher urinary aromatic amine levels than users of e-cigarettes, heated tobacco products, or nicotine replacement therapy, among whom levels did not differ from those of non-users (Scherer et al., 2022). Exposure to waterpipe tobacco also increases aromatic amine biomarkers (Yan et al., 2024). Epidemiological data indicate that approximately 50% of cases of bladder cancer are linked to smoking, with aromatic amines considered key carcinogens (Rozanec and Secin, 2020; Bellamri et al., 2022). A study integrating transcriptomic, genomic, and microenvironmental data identified 33 tobacco-related genes and developed a prognostic model in which high-risk patients had significantly shorter overall survival (Wu et al., 2024). Furthermore, 4-aminobiphenyl DNA adducts have been detected in 29% of non-tumoral bladder tissues, and human bladder epithelial cells (RT4) can bioactivate this compound via CYP2A to form adducts (Dobaradaran et al., 2022; Bellamri et al., 2019). Collectively, tobacco exposure is the primary non-occupational route of aromatic amine exposure in the general population and is closely associated with bladder cancer risk; therefore, tobacco control remains a key intervention to reduce this burden.

### 2.3 Dietary aromatic amines and the risk of bladder cancer

Dietary aromatic amines are heterocyclic amines (HCAs) formed during the high-temperature cooking of meat, such as barbecuing, frying, or roasting (Sivasubramanian et al., 2023; Guo et al., 2018). Epidemiological studies associate HCAs with increased bladder cancer risk (Guo et al., 2018). NHANES data showed that smokers had 40-fold higher urinary AαC levels than non-smokers; by contrast, even high intake of well-done beef increased urinary AαC by only 0.99 pg/mL, whereas vegetable intake reduced it, indicating that diet is a secondary but still significant source (Zhang et al., 2020). *In vitro*, HCAs such as AαC can be bioactivated by CYP1A in human bladder epithelial cells to form DNA adducts (Bellamri et al., 2019). However, in 41 bladder cancer patients, only 4-aminobiphenyl DNA adducts were detected, not those of typical HCAs, suggesting differential *in vivo* handling of various aromatic amines (Guo et al., 2018). Although dietary exposure levels are generally lower than those from tobacco sources, long-term frequent consumption of high-temperature processed meat may still pose a significant risk. Currently, high-quality prospective cohort studies directly validating the dose-response relationship between dietary HCA intake and bladder cancer incidence are lacking.

## 3 Recent advances in research on the core pathogenic mechanisms of aromatic amines in bladder cancer

Recent mechanistic studies have substantially advanced our understanding of how aromatic amines drive bladder carcinogenesis. The emerging paradigm includes not only classic metabolic activation and DNA adduction but also non-genotoxic events: oxidative stress, sustained DDR dysregulation, epigenetic alterations, and oncogenic signaling activation. Together, these advances integrate the multistep process from exposure to malignant transformation (Figure 1).

**FIGURE 1:**
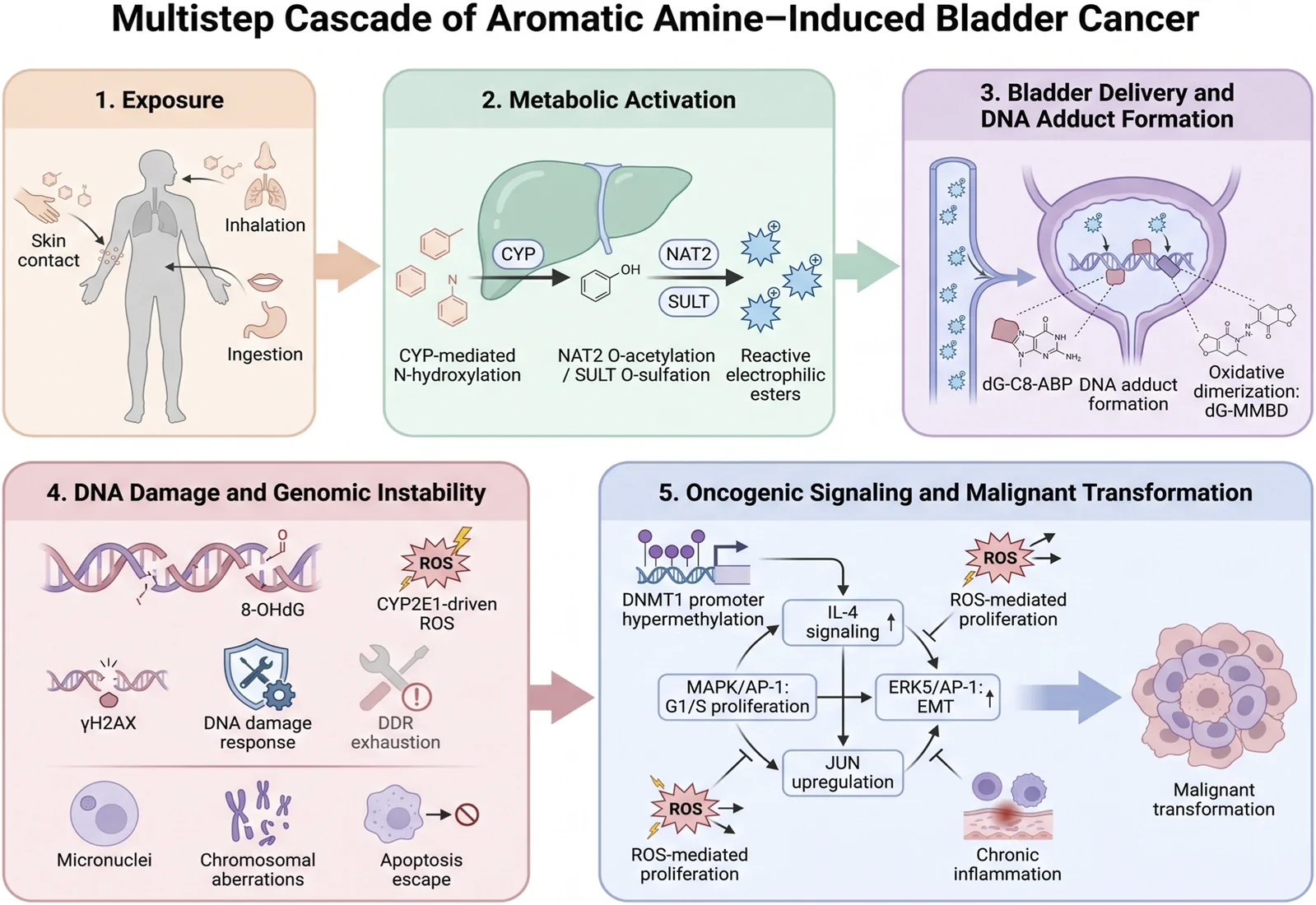
Multistep pathogenic cascade of aromatic amine–induced bladder cancer. This schematic depicts the integrated sequence of molecular events from exposure to malignant transformation. Aromatic amines enter the body via dermal, inhalation, or ingestion routes and undergo hepatic Phase I (CYP-mediated N-hydroxylation) and Phase II (NAT2-catalyzed O-acetylation or SULT-mediated O-sulfation) metabolism, generating highly reactive electrophilic ester intermediates. These metabolites are delivered through the bloodstream to the bladder, where they covalently bind to DNA, forming characteristic adducts (e.g., dG-C8-4-ABP). Concomitantly, CYP2E1-driven ROS production and oxidative dimerization of monocyclic amines (e.g., dG-MMBD) amplify genomic instability. The resulting DNA lesions—including double-strand breaks (γ-H2AX) and oxidative adducts (8-OHdG)—trigger the DNA damage response (DDR); however, chronic damage exhausts DDR capacity, leading to micronuclei, chromosomal aberrations, and apoptosis evasion. Epigenetic changes, such as DNMT1-mediated promoter hypermethylation, further cooperate with genotoxicity. These insults activate oncogenic pathways—MAPK/AP-1 (driving G1/S proliferation), ERK5/AP-1 (inducing EMT), JUN upregulation, and IL-4 signaling—which, together with ROS-mediated proliferation and chronic inflammation, orchestrate malignant transformation. NADPH oxidase and antioxidant interventions block these effects, highlighting oxidative stress as a central node. This integrated cascade mechanistically links exposure, genetic/epigenetic damage, and bladder carcinogenesis.

To systematically assess aromatic amine-induced toxicity, the field relies on a multi-tiered methodological framework encompassing *in vitro*, *in vivo*, and human biomonitoring approaches at the molecular, cellular, and tissue levels. At the molecular level, ultra-performance liquid chromatography-tandem mass spectrometry (UPLC-MS/MS) enables sensitive and specific quantification of DNA adducts—such as the dG-C8-4-ABP adduct of 4-aminobiphenyl—in both fresh-frozen and formalin-fixed paraffin-embedded (FFPE) tissues, with a detection limit of approximately one adduct per 10^9^ nucleotides (Guo et al., 2016; Mastrangelo et al., 2017). Oxidative DNA damage is concurrently assessed by measuring 8-hydroxy-2-deoxyguanosine (8-OHdG), while γ-H2AX foci formation serves as a sensitive surrogate marker for DNA double-strand breaks (Qi et al., 2020; Suzuki et al., 2023). At the cellular level, human urothelial cell lines (e.g., RT4, SV-HUC-1) are employed to evaluate the bioactivation capacity of specific aromatic amines via endogenous CYP enzymes (CYP2A, CYP1A) and to determine the relative genotoxic potency of different compounds through dose-response and time-course experiments (Bellamri et al., 2019; Qi et al., 2020). At the tissue and organism level, rodent models—particularly F344 rats and humanized-liver mice—replicate the *in vivo* metabolism and bladder-specific toxicity of aromatic amines, enabling histopathological evaluation of urothelial hyperplasia, dysplasia, and tumorigenesis alongside molecular endpoint measurements (Suzuki et al., 2025; Toyoda et al., 2023; Toyoda et al., 2019). In human populations, biomonitoring strategies integrate urinary metabolite profiling, DNA adduct detection in exfoliated bladder cells, and genotyping of metabolic enzyme polymorphisms (e.g., NAT2, NQO1, SULT1A1) to capture internal exposure doses, early biological effects, and individual susceptibility, thereby providing a direct bridge between experimental toxicology and epidemiological risk assessment (Guo et al., 2018; Mastrangelo et al., 2017; Yun et al., 2018; Wang et al., 2017). These complementary approaches—from targeted chemical analysis to functional cellular assays and population-based molecular epidemiology—constitute the current methodological arsenal for assessing aromatic amine-induced bladder toxicity and for establishing exposure-effect relationships.

### 3.1 Metabolic activation: Phase I and phase II enzymes and species differences

The carcinogenic effects of aromatic amine compounds depend on their metabolic activation *in vivo*. Phase I and Phase II drug-metabolizing enzyme systems mediate this process, involving the synergistic action of the liver, bladder epithelial cells, and the gut microbiota (Pan et al., 2025). In the activation pathway, CYP enzymes first catalyze N-hydroxylation of typical aromatic amines, yielding N-hydroxyaromatic amines (Tsukino et al., 2004; Shimada, 2017; Baldauf et al., 2020). CYP2A and CYP1A, which are endogenously expressed in the bladder uroepithelium, can directly activate 4-ABP and AαC into DNA adducts, with 4-ABP adduct levels being ten times higher than those of AαC; inhibition of CYP2A can reduce adduct levels by 90% (Bellamri et al., 2019). The human bladder epithelial cell line RT4 can directly metabolize 4-ABP and AαC; CYP2A plays a dominant role in the activation of 4-ABP, while CYP1A is primarily involved in the activation of AαC (Bellamri et al., 2019). O-toluidine is primarily metabolized by CYP3A4 in human hepatocytes, whereas in mice it is metabolized by CYP2C29, suggesting significant differences in metabolic pathways between species (Yanagiba et al., 2024). Some monocyclic aromatic amines can generate semidine-type dimers with strong cytotoxicity and mutagenicity via the S9 complex; among these, dimers containing the o-anisidine structure undergo faster metabolic conversion and exhibit greater toxicity (Pan et al., 2025). Furthermore, precursor compounds such as acetoacetyl-o-toluidine can be hydrolyzed *in vivo* to o-toluidine (OTD), which, as an active metabolite, directly induces bladder epithelial hyperplasia and DNA damage, indicating that metabolic release is a key step in its carcinogenic activity (Pan et al., 2025). During Phase II esterification, N-hydroxyaromatic amines undergo further O-acetylation catalyzed by NAT2 or O-sulfation catalyzed by SULT1A1/SULT1D1, generating highly reactive ester intermediates. These intermediates decompose into electrophilic nitroanion species that attack DNA, forming characteristic adducts (Tsukino et al., 2004; Baldauf et al., 2020; Li et al., 2018). Animal studies have revealed that androgens can suppress the expression of SULT1A1/SULT1D1 in the mouse liver, thereby decreasing the liver’s ability to detoxify 4-ABP. Consequently, more active metabolites are transported to the bladder, thereby exacerbating gender-specific susceptibility to bladder cancer (Li et al., 2018). The body limits aromatic amine toxicity through multiple metabolic detoxification systems. N-acetylation is the primary detoxification pathway: NAT2 catalyzes the formation of stable acetylated products from aromatic amines; in contrast, individuals with a slow acetylator phenotype exhibit significantly reduced enzyme activity, and the risk of bladder cancer among current smokers increases to an HR of 5.70 (Yuliwulandari et al., 2019; Salazar-González et al., 2020; Pradhan et al., 2024; Hein and Doll, 2017). These findings have significant clinical and preventive implications.

### 3.2 DNA adduct formation and genotoxic consequences

The core mechanism by which aromatic amine compounds induce bladder cancer lies in their metabolic activation, followed by covalent binding to DNA to form specific adducts, ultimately leading to genotoxic damage. Direct evidence indicates that 4-aminobiphenyl (4-ABP)-related adducts are the most prevalent aromatic amine-derived DNA lesions in human bladder tissue. In non-tumor bladder specimens from cancer patients, the deoxyguanosine C8 adduct N-(dG-C8)-4-ABP was detectable in 29% of individuals, at levels ranging from 1.4 to 33.8 adducts per 10^9^ nucleotides, whereas adducts derived from other aromatic amines and lipid peroxidation remained below the detection limit (Bellamri et al., 2019; Guo et al., 2018). Notably, this adduct can be recovered not only from fresh tissue but also from formalin-fixed, paraffin-embedded (FFPE) archival specimens, with recovery rates comparable to those from fresh-frozen tissue, thereby offering a valuable tool for retrospective molecular epidemiological investigations (Guo et al., 2016; Mastrangelo et al., 2017). In addition, the dG-C8 adduct remains stable in exfoliated urinary cells for up to 24 h, supporting its utility as a non-invasive biomarker (Yun et al., 2018). Beyond the formation of monoamine-derived adducts, dimeric metabolites of monocyclic aromatic amines generated under oxidative conditions can react with DNA to produce dG-MMBD adducts, which have been detected in both rat liver DNA and in bovine thymus DNA and exhibit enhanced cytotoxicity and mutagenicity (Tajima et al., 2020; Kobayashi et al., 2022; Yokota et al., 2023). Concurrently, hepatic cytochrome P450-mediated N-hydroxylation, followed by O-acetylation catalyzed by N-acetyltransferase 2 (NAT2), generates highly reactive ester intermediates that are transported via the bloodstream to the bladder. A humanized liver mouse model further confirmed that o-toluidine metabolites accumulate in urine, accompanied by bladder epithelial proliferation and DNA damage (Suzuki et al., 2025).

The downstream genotoxic effects of these adducts are multifaceted. Direct evidence from experimental models indicates that certain aromatic amines can induce DNA double-strand breaks (DSBs), as detected by γ-H2AX foci formation. In human urothelial cell lines, comparative exposure to six aromatic amines revealed varying potencies in γ-H2AX induction, with o-chloroaniline and 2,4-dimethylaniline exhibiting the most pronounced genotoxic effects (Qi et al., 2020). Similarly, in the bladder epithelium of male F344 rats, o-toluidine exposure led to sustained γ-H2AX elevation that correlated with progressive hyperplasia and dysplasia, whereas o-anisidine induced only transient γ-H2AX expression without overt histopathological changes, suggesting subclinical genotoxic damage (Toyoda et al., 2023; Toyoda et al., 2019). Notably, these γ-H2AX responses were observed under conditions where oxidative stress was not the primary driver, implying a direct or proximate genotoxic effect of the parent compounds or their metabolites.

Alongside direct genotoxicity, aromatic amines also promote DSBs indirectly through oxidative stress. Exposure to these compounds promotes the formation of 8-hydroxy-2′-deoxyguanosine (8-OHdG), an oxidative DNA adduct, in the bladder epithelium, indicating substantial ROS generation (Suzuki et al., 2023). Mechanistically, 2,4-dimethylaniline induces γ-H2AX expression through CYP2E1-mediated production of ROS, an effect that can be abrogated by CYP2E1 inhibitors or antioxidants, confirming that oxidative metabolism is a key upstream event (Suzuki et al., 2023). Importantly, treatment with an NADPH oxidase inhibitor or the antioxidant N-acetylcysteine reduces Ki67, γ-H2AX, and 8-OHdG levels in a concentration-dependent manner, underscoring the critical role of oxidative stress in mediating genotoxicity and the proliferative response (Suzuki et al., 2023). These findings suggest that a substantial portion of DSBs observed following aromatic amine exposure may represent downstream consequences of oxidative DNA damage rather than direct adduct-mediated strand cleavage.

The current evidence supports a dual mechanism for DSB induction by aromatic amines: a direct genotoxic component likely mediated by specific DNA adducts that interfere with replication and repair, and an indirect component driven by ROS-induced oxidative damage. However, most direct evidence for DSB formation comes from *in vitro* and rodent models. In human bladder tissues, the relative contributions of direct versus oxidative pathways remain to be definitively quantified. The detection of γ-H2AX in human biomonitoring studies should therefore be interpreted as an integrated marker of genotoxic stress rather than as a specific indicator of direct adduct-induced strand breaks.

In terms of mutagenic consequences, aromatic amine metabolites can form multi-site adducts with deoxyguanosine, distorting the DNA double helix and interfering with normal replication and repair processes (Matsui et al., 2020). Persistent DNA adducts and DSBs activate the DNA damage response (DDR). However, chronic damage may exhaust the DDR capacity, allowing damaged cells to evade apoptosis, accumulate mutations, and eventually develop micronuclei, chromosomal breaks, and aneuploidy (Wohlfahrt et al., 2024). Furthermore, aromatic amine exposure upregulates DNMT1, leading to promoter hypermethylation, DNMT1 inhibition reduces DNA adduct levels, suggesting epigenetic cooperation with genotoxicity (Xu et al., 2025). Single-cell transcriptomic analysis further reveals that 4-ABP reprograms fibroblasts and mast cells and enriches IL-4 signaling, indicating that chronic inflammation may amplify the genomic damage induced by aromatic amines (Xu et al., 2025). In summary, DNA adducts formed by aromatic amine metabolites distort DNA structure, interfere with replication and repair, and induce mutations and chromosomal aberrations. Oxidative stress and dimer metabolites further exacerbate these genotoxic effects, collectively contributing to the carcinogenic process in the bladder.

### 3.3 Differential toxicity profiles among aromatic amines

Existing evidence clearly indicates that different aromatic amines exhibit substantially distinct toxicity profiles, with variations across multiple dimensions—metabolic activation pathways, DNA damage spectra, cellular responses, and *in vivo* pathological outcomes. These differences have critical implications for risk assessment and regulatory prioritization.

At the metabolic level, structural variations among aromatic amines dictate their preferential processing by specific cytochrome P450 isoforms. For instance, 4-aminobiphenyl (4-ABP) is predominantly bioactivated by CYP2A in human bladder epithelial cells, whereas the heterocyclic amine AαC is primarily activated by CYP1A, with 4-ABP adduct formation being tenfold higher than that of AαC (Bellamri et al., 2019). Similarly, CYP3A4 metabolizes o-toluidine in human hepatocytes, whereas CYP2C29 does so in mice, indicating that species differences in CYP expression further complicate cross-species extrapolation of toxicity data (Yanagiba et al., 2024). During Phase II metabolism, NAT2-mediated O-acetylation and SULT1A1/SULT1D1-mediated O-sulfation also exhibit substrate specificity, generating varying quantities of reactive ester intermediates (Tsukino et al., 2004; Baldauf et al., 2020; Li et al., 2018). Furthermore, certain monocyclic aromatic amines, such as o-anisidine, undergo more rapid oxidative dimerization to form semidine-type dimers with enhanced cytotoxicity and mutagenicity, while others produce fewer or less toxic dimeric metabolites (Pan et al., 2025).

At the genotoxic level, quantitative and qualitative differences in DNA damage are well documented. Comparative γ-H2AX analysis in human urothelial cells revealed that o-chloroaniline and 2,4-dimethylaniline are the most potent inducers of DNA double-strand breaks among six tested aromatic amines, whereas others exhibited substantially lower genotoxic potency (Qi et al., 2020). In human bladder tissues, only 4-ABP-derived dG-C8 adducts are consistently detectable, while adducts from 2-naphthylamine, 2-methylaniline, and 2,6-dimethylaniline remain below the limit of quantification, suggesting differential *in vivo* adduct formation efficiency and/or differential DNA repair kinetics (Guo et al., 2018).

At the histopathological level, distinct outcomes are observed even among structurally similar compounds. In the F344 rat model, o-toluidine exposure leads to progressive urothelial hyperplasia, dysplasia, and significant DNA damage (γ-H2AX elevation), whereas o-anisidine induces only mild mucosal hyperplasia accompanied by inflammation, without the same degree of DNA damage or progressive lesions (Toyoda et al., 2019). Moreover, high-dose exposure to certain aromatic amines produces marked bladder toxicity, while structurally related compounds do not elicit comparable effects, underscoring the critical role of subtle structural differences (Toyoda et al., 2023).

At the epidemiological level, these differential toxicities translate into markedly different cancer risks in human populations. In Japanese chemical workers, o-toluidine exposure yielded a standardized incidence ratio (SIR) of 56.8 with a clear dose-response relationship, whereas other aromatic amines elevated the SIR but showed no significant dose-response trend (Nakano et al., 2021). This epidemiological heterogeneity aligns with the experimental findings described above and suggests that o-toluidine may possess uniquely potent bladder carcinogenicity among the aromatic amines.

These lines of evidence demonstrate that the toxicity profile of aromatic amines is not uniform but is heavily influenced by structural features—particularly the type, number, and position of substituents on the aromatic ring, as well as the compound’s lipophilicity and redox potential, which collectively determine its metabolic fate, DNA reactivity, and downstream pathological consequences. This heterogeneity poses a significant challenge for quantitative risk assessment and underscores the need for compound-specific rather than class-based regulatory approaches, as well as for further systematic structure-activity relationship studies to identify the key molecular determinants of carcinogenic potency.

### 3.4 Activation of oncogenic signaling pathways and malignant transformation

Aromatic amines hijack key signaling pathways to drive malignant transformation. In normal human bladder epithelial cells, benzidine activates the MAPK/AP-1 pathway, promoting the G1/S transition and proliferation; MAPK inhibitors block this effect (Zhao et al., 2018). In bladder cancer cells, benzidine also activates the ERK5/AP-1 axis, inducing epithelial-mesenchymal transition (EMT), which is reversed by ERK5 inhibition or curcumin (Liu et al., 2017; Sun et al., 2018). In a rat model, acetoacetyl-o-toluidine (AAOT) upregulates JUN and its targets (Yukimatsu et al., 2019). Additionally, 4-ABP triggers IL-4 signaling, with HSP90B1 and IL4R as high-affinity binding partners (Xu et al., 2025). Oxidative stress further activates proliferation-associated pathways; inhibition of NADPH oxidase reduces Ki67, γ-H2AX, and 8-OHdG, confirming that ROS both damage DNA and promote proliferation (Nakano et al., 2025; Suzuki et al., 2023). In aromatic amine-induced bladder cancer, key signaling pathways and regulatory factors act synergistically to regulate cell proliferation, epithelial-mesenchymal transition, DNA damage response, and metabolic activation.

## 4 Research on susceptibility markers and risk prediction models

Genetic polymorphisms significantly influence individual susceptibility to aromatic amine-induced bladder cancer. The NAT2 slow acetylator phenotype is a well-established risk factor. In Iranian slow acetylators exposed to β-naphthylamine, HPRT mutations, DSBs, and ROS were elevated (Habil et al., 2022). A United Kingdom Biobank cohort further showed that current smokers with slow NAT2 have a 5.70-fold higher risk (Pradhan et al., 2024). Beyond NAT, NQO1 and SULT1A1 polymorphisms synergize with smoking, elevating risk up to 3.7-fold and 2.4-fold, respectively (Wang et al., 2017). Conversely, a rare UGT1A variant enhances carcinogen clearance and protects against bladder cancer (Tang et al., 2012). Researchers have incorporated these susceptibility markers into risk prediction models. A model integrating clinical, laboratory, and genetic factors achieved AUCs of 0.93 (full) and 0.86 (simplified) (Mastrangelo et al., 2017). A tobacco-related gene prognostic model from the Nanjing cohort showed an HR of 2.25 for poor survival (Wu et al., 2024). Single-cell RNA sequencing identified HSP90B1 and IL4R as high-affinity 4-ABP binding partners, suggesting their potential as susceptibility biomarkers (Xu et al., 2025). Thus, combining internal exposure and genetic markers enables individualized risk assessment for high-risk populations.

## 5 Limitations, controversies, and unresolved issues in current research

Current research controversies primarily center on the uncertainty regarding the risks of low-dose combined exposure. In real-world settings, populations are often simultaneously exposed to multiple aromatic amines and other coexisting substances, yet systematic data on synergistic effects are lacking. At the same time, no unified standard exists for quantitatively comparing the carcinogenic potency of different aromatic amines, and both experimental and epidemiological studies indicate significant structural heterogeneity. Furthermore, control for confounding factors in population-based studies is generally inadequate; key variables such as diet and genetics are often not sufficiently adjusted for, and exposure assessments rely primarily on questionnaires or occupational history rather than direct biomonitoring, leading to misclassification bias that further undermines the reliability of causal inferences.

### 5.1 Controversy surrounding the health risk assessment of low-dose combined exposure

The impact of low-dose combined exposure to aromatic amines on bladder cancer risk remains controversial. Although trace levels of monocyclic amines (e.g., o-toluidine) are detected in tobacco smoke, it is unclear whether such low concentrations induce significant biological effects (Konorev et al., 2023). Moreover, in tobacco smoke condensates at <1% cigarette concentration, non-amine components may be more genotoxic than 4-aminobiphenyl or 2-naphthylamine, calling into question the relative contribution of aromatic amines in co-exposure scenarios (Bellamri et al., 2022). Complicating the issue, monocyclic amines can dimerize *in vivo*, forming metabolites with greater mutagenic and cytotoxic potential that may amplify carcinogenic risk, especially under combined exposure (Kobayashi et al., 2022; Kobayashi et al., 2021). However, systematic data on low-dose multi-amine interactions are lacking, hindering accurate risk assessment for real-world exposures.

### 5.2 Factors synergizing with aromatic amine carcinogenicity

Beyond the intrinsic toxicity of aromatic amines themselves, accumulating evidence indicates that several exogenous and endogenous factors can synergistically amplify their carcinogenic effects. These synergistic interactions occur at multiple levels—from increasing internal exposure doses, through modulating metabolic activation and detoxification, to exacerbating DNA damage and inflammatory responses.

Tobacco smoking represents the most well-established synergistic factor. Tobacco smoke is not only a direct source of aromatic amines but also contains numerous other carcinogens and co-carcinogens that may interact with aromatic amines to potentiate bladder carcinogenesis (Konorev et al., 2023; Scherer et al., 2022; Bellamri et al., 2022). The synergistic effect is particularly evident at the genetic level: smokers carrying the NAT2 slow acetylator phenotype exhibit a dramatically elevated bladder cancer risk, with a hazard ratio of 5.70 compared to non-smokers with rapid acetylation (Pradhan et al., 2024). Similarly, polymorphisms in NQO1 and SULT1A1 synergize with smoking to increase bladder cancer risk by 3.7-fold and 2.4-fold, respectively (Wang et al., 2017). This gene–environment synergy is mechanistically grounded in the dual role of smoking—providing both the carcinogenic substrate and modulating the host metabolic capacity to activate them.

Genetic polymorphisms in metabolic enzymes act as independent susceptibility factors that can synergize with aromatic amine exposure. Beyond the well-characterized NAT2 slow acetylator phenotype, which reduces detoxification and increases reactive N-hydroxy metabolite levels (Yuliwulandari et al., 2019; Salazar-González et al., 2020; Pradhan et al., 2024; Hein and Doll, 2017), polymorphisms in CYP1A2, SULT1A1, and NQO1 have been shown to modulate individual capacity for aromatic amine activation or detoxification (Wang et al., 2017; Tsukino et al., 2004). Individuals carrying multiple “high-risk” genotypes may experience substantially enhanced carcinogen bioactivation and DNA adduct formation, representing a polygenic synergistic effect.

Oxidative stress and chronic inflammation constitute another critical synergistic axis. Aromatic amine exposure itself generates ROS through CYP2E1-mediated metabolism, leading to oxidative DNA damage and double-strand breaks (Suzuki et al., 2023). When combined with pre-existing or concurrent inflammatory conditions—such as those induced by other environmental pollutants, chronic infections, or metabolic disorders—the oxidative burden is amplified, potentially overwhelming DNA repair capacity and promoting mutagenesis (Wohlfahrt et al., 2024; Xu et al., 2025). Single-cell transcriptomic data further reveal that 4-aminobiphenyl reprograms fibroblasts and mast cells and enriches IL-4 signaling, indicating that aromatic amines can actively remodel the tissue microenvironment toward a pro-inflammatory, pro-carcinogenic state that synergizes with other inflammatory stimuli (Xu et al., 2025).

Co-exposure to other chemicals in occupational or environmental settings represents a less characterized but potentially significant synergistic domain. In tobacco smoke condensate, non-amine components—such as hydroquinone and cresol—contribute substantially to cytotoxicity and apurinic/apyrimidinic site formation, with a mixture of hydroquinone and cresol reproducing most of the genotoxic effects induced by whole smoke condensate (Bellamri et al., 2022). This suggests that aromatic amines may act in concert with other smoke constituents to produce additive or synergistic DNA damage. Similarly, in occupational settings, workers are often exposed to complex mixtures of aromatic amines, solvents, and other industrial chemicals, yet systematic data on their interactive effects remain limited (Hosseini et al., 2023; Nakano et al., 2021).

Hormonal factors, particularly androgens, may also synergize with aromatic amine carcinogenicity through metabolic modulation. Animal studies have shown that androgens suppress SULT1A1/SULT1D1 expression in the mouse liver, reducing hepatic detoxification capacity for 4-aminobiphenyl. This leads to a greater proportion of active metabolites being transported to the bladder, potentially explaining the higher bladder cancer incidence in males (Li et al., 2018). This hormone–carcinogen interaction suggests a biological basis for sex differences in susceptibility that warrants further investigation.

Collectively, the synergistic effects between aromatic amines and the factors discussed above—tobacco smoke, genetic polymorphisms, oxidative/inflammatory conditions, co-exposure to other chemicals, and hormonal milieu—operate through distinct yet interconnected mechanisms: increasing substrate availability, modulating metabolic activation, impairing detoxification, exacerbating DNA damage, and creating a permissive microenvironment for malignant transformation. Understanding these interactions is essential for accurate risk stratification, particularly in high-risk occupational populations where multiple factors often coincide, and for designing effective multi-target prevention strategies.

### 5.3 Factors conferring antagonistic effects against aromatic amine carcinogenicity

Beyond the synergistic factors that amplify aromatic amine toxicity, accumulating evidence also points to several endogenous and exogenous factors that can antagonize their carcinogenic effects. These antagonistic factors operate through diverse mechanisms—enhancing carcinogen detoxification and elimination, directly scavenging reactive intermediates, blocking metabolic activation, or interfering with oncogenic signaling pathways. Identifying and understanding these protective factors is of substantial translational value, as they may inform chemopreventive strategies for high-risk populations.

Endogenous protective factors: genetic determinants of enhanced detoxification. At the genetic level, certain polymorphisms in metabolic enzymes confer resistance to aromatic amine-induced carcinogenesis. While the NAT2 slow acetylator phenotype is a well-established risk factor, the rapid acetylator phenotype—characterized by high NAT2 enzyme activity—accelerates the N-acetylation of aromatic amines, promoting their conversion to stable, excretable metabolites and thereby reducing the availability of reactive intermediates for DNA adduct formation (Yuliwulandari et al., 2019; Salazar-González et al., 2020; Pradhan et al., 2024; Hein and Doll, 2017). Similarly, a rare coding variant in the UGT1A locus has been identified that enhances the glucuronidation and clearance of carcinogenic aromatic metabolites, conferring a protective effect against bladder cancer (Tang et al., 2012). These genetic protective factors are particularly relevant in the context of gene–environment interactions: individuals carrying such protective alleles may have substantially lower susceptibility even when exposed to comparable levels of aromatic amines.

Exogenous protective factors: natural products with chemopreventive potential. Several naturally occurring compounds have demonstrated antagonistic effects against aromatic amine-induced carcinogenesis in experimental models. Curcumin, a polyphenolic compound derived from turmeric, has been shown to reverse benzidine-induced EMT by suppressing the ERK5/AP-1 signaling pathway in human urothelial cells (Liu et al., 2017). This effect is mechanistically significant, as ERK5/AP-1 activation is a key driver of aromatic amine-induced malignant transformation. Genistein, an isoflavone found in soybeans, has been demonstrated to inhibit tyrosine kinase activity and downstream signaling cascades, potentially counteracting aromatic amine-induced proliferative signals (Toyoda et al., 2023). N-acetylcysteine (NAC), a widely available antioxidant, has been shown to reduce γ-H2AX, 8-OHdG, and Ki67 levels in a concentration-dependent manner in bladder epithelial cells exposed to aromatic amines, thereby alleviating bladder hyperplasia and DNA damage (Suzuki et al., 2023). The chemopreventive potential of these natural products is further supported by their favorable safety profiles, making them attractive candidates for dietary intervention or adjunctive therapy in high-risk populations.

Exogenous protective factors: synthetic compounds and pharmacological inhibitors. Synthetic chemicals that modulate aromatic amine metabolism or signaling also exhibit antagonistic effects. CYP enzyme inhibitors represent a mechanistically well-defined class of antagonists. For instance, inhibition of CYP2A, the primary enzyme responsible for 4-aminobiphenyl bioactivation in bladder epithelial cells, can reduce DNA adduct formation by up to 90% (Bellamri et al., 2019). CYP2E1 inhibitors have been shown to abrogate 2,4-dimethylaniline-induced γ-H2AX expression, confirming that blocking the metabolic activation pathway effectively antagonizes genotoxicity (Suzuki et al., 2023). MAPK inhibitors directly interfere with the downstream oncogenic signaling cascade: they block benzidine-induced MAPK/AP-1 activation, thereby suppressing the G1/S transition and abnormal cell proliferation (Zhao et al., 2018). NADPH oxidase inhibitors reduce ROS production, thereby decreasing oxidative DNA damage and limiting the proliferative drive induced by aromatic amine exposure (Suzuki et al., 2023). These pharmacological agents, while currently used primarily as experimental tools, may offer future therapeutic or chemopreventive applications, particularly for individuals with established DNA damage or precancerous lesions.

Beyond specific compounds, broader dietary patterns may also modulate susceptibility to aromatic amine-induced bladder cancer. Although direct evidence remains limited, the association between vegetable intake and reduced urinary levels of heterocyclic aromatic amines suggests that plant-based diets may protect, possibly through the combined actions of antioxidants, fiber, and phytochemicals that enhance elimination or scavenge reactive intermediates (Zhang et al., 2020). However, high-quality prospective studies validating the protective effects of specific dietary patterns against aromatic amine-associated bladder cancer are currently lacking.

These antagonistic factors—whether genetic, natural product-derived, synthetic, or dietary—operate through a shared principle: they intercept the carcinogenic cascade at critical nodes, including metabolic activation, DNA damage propagation, oxidative stress amplification, and oncogenic signaling transduction. Understanding these protective mechanisms is essential not only for comprehensive risk assessment but also for rationally designing chemopreventive strategies targeting high-risk occupational populations. Identifying effective antagonists, particularly naturally occurring compounds with established safety profiles, represents a promising avenue for reducing the disease burden of aromatic amine-related bladder cancer.

### 5.4 The differences in carcinogenic potency among various types of aromatic amines have not yet been clearly established

Although many aromatic amines are established bladder carcinogens, their relative carcinogenic potency and structure-activity relationships remain incompletely understood. Epidemiological evidence shows substantial variation: in Japanese chemical workers, o-toluidine exposure yielded a standardized incidence ratio (SIR) of 56.8 with a clear dose-response relationship, whereas other aromatic amines elevated the SIR without a significant dose-response (Nakano et al., 2021). *In vitro*, different aromatic amines induce γ-H2AX expression in human urothelial cells to varying degrees, with o-chloroaniline and 2,4-dimethylaniline being the most genotoxic (Qi et al., 2020). Animal studies further confirm this heterogeneity: o-toluidine causes progressive bladder lesions and significant DNA damage, while o-anisidine induces only mild hyperplasia with inflammation (Toyoda et al., 2019). Additionally, certain aromatic amines exhibit marked bladder toxicity at high doses, whereas structurally similar compounds do not (Toyoda et al., 2023). Currently, a unified quantitative framework or cross-species comparison system for evaluating these differences in carcinogenic potency is lacking, hindering precise risk stratification and regulatory strategies.

### 5.5 Limitations of confounder control in population-based studies

Human epidemiological studies on aromatic amines and bladder cancer often inadequately control confounding factors. Although some studies adjust for smoking, age, and race (Hosseini et al., 2023; Oliver et al., 2024). Potential confounders, including dietary habits, concurrent environmental exposures, gut microbiota, medication use, and genetic background, are often not fully incorporated (Pan et al., 2025; Mikhaleva et al., 2021). For example, an Iranian case-control study controlled for smoking and opium use but did not assess other occupational or environmental exposures (Hosseini et al., 2023). A South African study identified Black race, male sex, and age as risk factors but did not explore specific aromatic amine exposure mechanisms or confounding biases (Oliver et al., 2024). Furthermore, exposure levels are typically estimated from work histories or questionnaires rather than direct biomonitoring, leading to significant exposure misclassification (Nakano et al., 2021). Even when DNA adducts are used as internal exposure markers, detection rates remain low; for instance, adducts of 2-naphthylamine, 2-methylaniline, and 2,6-dimethylaniline in human bladder DNA were all below the limit of quantification, further undermining the reliability of exposure-effect relationships (Guo et al., 2018). These methodological limitations introduce uncertainty into causal inference and risk quantification.

## 6 Future research directions and application prospects

Future research should integrate multi-omics data to construct a complete molecular causal chain from aromatic amine exposure to malignant transformation, promote personalized prevention strategies, conduct stratified screening of high-risk occupational populations based on metabolic enzyme genotypes, and implement dynamic early warning systems through biomonitoring. In addition, researchers should explore targeted interventions for early-stage DNA damage, ultimately establishing a closed-loop prevention and control system that covers the entire “monitoring–early warning–intervention” chain (Figure 2).

**FIGURE 2:**
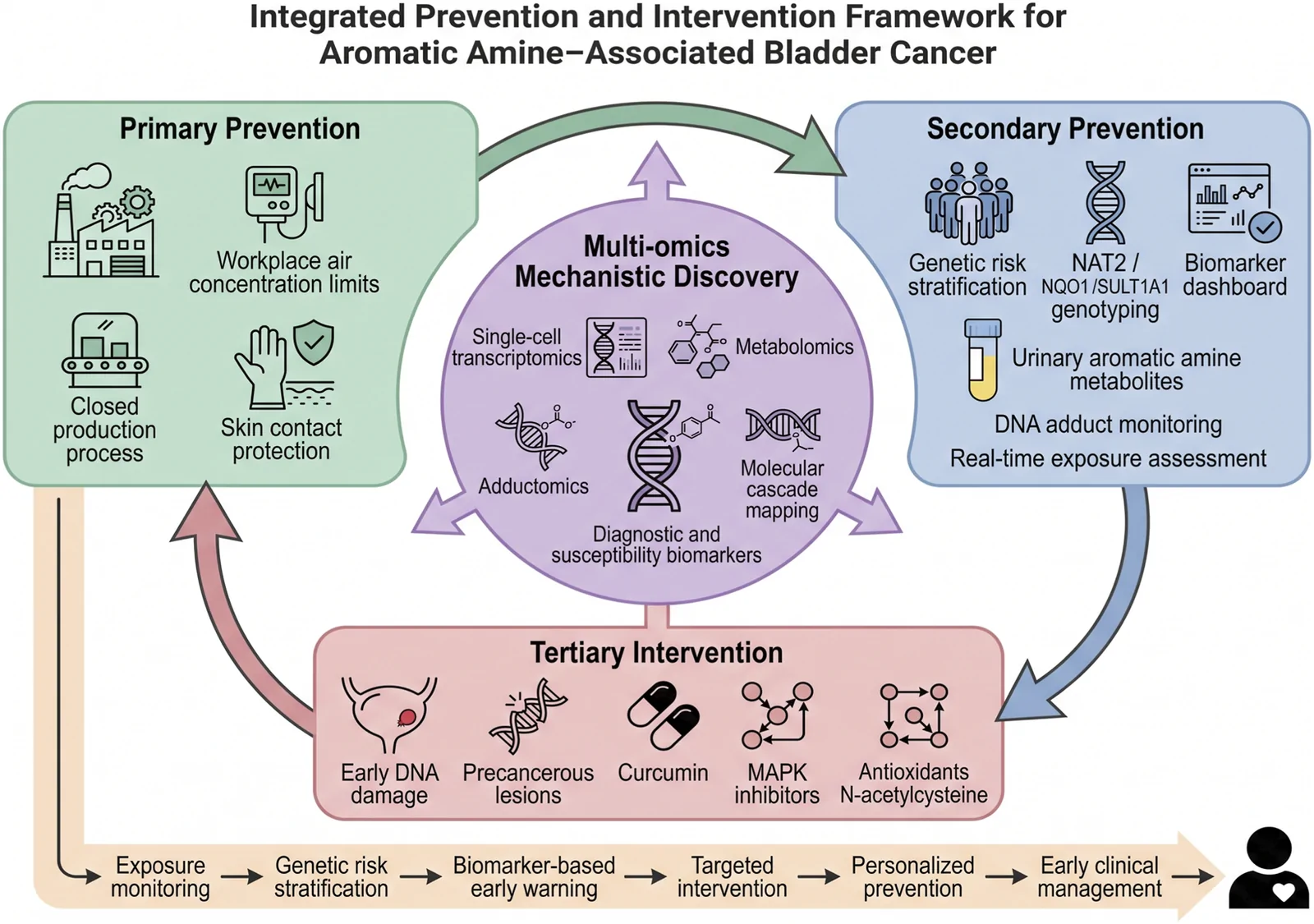
Integrated framework for future prevention and intervention strategies against aromatic amine–related bladder cancer. This schematic outlines a closed-loop “monitoring–early warning–intervention” system designed to reduce the disease burden. At the primary prevention level, strict workplace air limits, enclosed production processes, and prioritized skin-contact protection are implemented. Secondary prevention incorporates metabolic enzyme genotyping (e.g., NAT2, NQO1, SULT1A1) for stratified screening of high-risk occupational populations, combined with dynamic biomonitoring of urinary aromatic amine metabolites and DNA adducts for real-time exposure assessment. Tertiary intervention targets individuals with early DNA damage or precancerous lesions, using pharmacological agents such as curcumin, MAPK inhibitors, and antioxidants (e.g., N-acetylcysteine). The framework leverages multi-omics data (single-cell transcriptomics, metabolomics, adductomics) to elucidate the complete molecular cascade and identify diagnostic/susceptibility biomarkers. Continuous feedback from exposure surveillance, genetic risk stratification, biomarker-based warning, and targeted intervention enables personalized prevention and early clinical management, translating mechanistic insights into actionable strategies for high-risk populations.

### 6.1 Applications of multi-omics technologies in mechanism elucidation

Multi-omics integration has become a powerful tool for dissecting the molecular mechanisms of aromatic amine-induced bladder cancer. Single-cell transcriptomics revealed specific gene expression profiles in fibroblast and mast cell subpopulations after 4-aminobiphenyl (4-ABP) exposure, identifying 15 functionally conserved genes associated with the IL-4 signaling pathway. Further machine learning identified six core diagnostic genes, including HSP90B1 and IL4R, which were confirmed to bind 4-ABP with high affinity, suggesting regulatory roles in carcinogenesis (Xu et al., 2025). Multi-omics integration using the Nanjing Bladder Cancer Cohort and TCGA databases established a prognostic scoring model for tobacco carcinogen-associated genes and revealed crosstalk among cancer-associated fibroblasts (CAFs), epithelial-mesenchymal transition (EMT), and immune evasion, providing new insights into tumor microenvironment remodeling (Wu et al., 2024). Metabolomics also plays a key role. In tobacco smoke condensate (TSC) experiments, metabolomic analysis showed that the acidic and neutral components of TSC alter the glutathione (GSH) biosynthetic pathway and generate more than 40 GSH adducts, with hydroquinone adducts being the most abundant. A mixture of hydroquinone and cresol reproduced most of the cytotoxicity and apurine/apyrimidine (AP) site formation induced by TSC, indicating that metabolomics helps identify key toxic mediators (Bellamri et al., 2022). Concurrently, DNA adductomics in rat models detected oxidative adducts, such as 8-OHdG, in bladder tissue after exposure to o-toluidine or acetoacetic acid-o-toluidine; together with NADPH oxidase inhibition experiments, this validated the role of oxidative stress in aromatic amine-induced bladder cancer (Suzuki et al., 2023). In the future, integrating multi-level data will help establish a complete causal chain linking aromatic amine exposure, metabolic activation, DNA damage, signaling-pathway activation, and malignant transformation, thereby advancing precise etiological stratification and personalized risk assessment.

### 6.2 Protection strategies and clinical intervention approaches for occupational exposure to aromatic amines

Occupational exposure to aromatic amines remains a major cause of bladder cancer, especially in dye, rubber, leather, textile, and organic chemical industries, where approximately 20% of urothelial bladder cancer cases are linked to such exposure (Alouini, 2024). Therefore, source control and personal protective measures are urgently needed. Workers exposed to o-toluidine have a standardized incidence ratio of 56.8, with a clear dose-response relationship, mandating strict workplace air limits, enclosed processes, and local exhaust ventilation (Nakano et al., 2021). Notably, six common aromatic amines achieve 70%–80% transdermal penetration within 8 h in human skin models; thus, in addition to respiratory protection, skin-contact measures should be prioritized (Nakano et al., 2025). Personalized prevention based on metabolic gene polymorphisms holds great potential. Smokers with NQO1 or SULT1A1 polymorphisms have up to a 3.71-fold higher bladder cancer risk (Wang et al., 2017). NAT2 slow acetylators, especially those carrying the Asian-prevalent allele NAT2*7B, exhibit increased genotoxicity and oxidative stress (Habil et al., 2022). Genotyping should therefore be incorporated into health monitoring for high-risk workers to enable enhanced follow-up and early screening. Future efforts could combine this with the detection of urinary aromatic amine metabolites to establish a closed-loop system that includes dynamic biomonitoring, risk warning, and intervention feedback. Targeted interventions are also promising for individuals with DNA damage or early lesions. Curcumin reverses diphenylamine-induced EMT by inhibiting ERK5/AP-1 signaling (Liu et al., 2017). MAPK inhibitors block aniline-induced MAPK/AP-1 activation, thereby suppressing abnormal proliferation (Zhao et al., 2018). Antioxidants reduce γ-H2AX, 8-OHdG, and Ki67 levels, thereby alleviating bladder hyperplasia and offering chemoprevention for high-risk groups (Suzuki et al., 2023). In summary, an integrated system of exposure monitoring, susceptibility screening, early intervention, and targeted protection should be established to reduce the disease burden of aromatic amine-related bladder cancer.

## 7 Conclusion

This review consolidates current evidence establishing aromatic amines as major determinants of bladder cancer. Epidemiological studies consistently implicate occupational sources—particularly in dye, rubber, and petrochemical industries—and tobacco smoke as the primary contributors to human exposure, with inhalation and dermal absorption representing the predominant routes of uptake. Dietary heterocyclic amines, while secondary to tobacco in quantitative terms, may pose additional risks through long-term consumption of high-temperature processed meats.

Mechanistically, the carcinogenic cascade begins with phase I and phase II metabolic activation, generating electrophilic intermediates that covalently bind to DNA to form characteristic adducts. These DNA lesions, together with oxidative stress-derived damage and induction of DNA double-strand breaks, drive genomic instability. Concurrent activation of oncogenic signaling pathways (MAPK/AP-1, ERK5/AP-1) promotes aberrant proliferation, epithelial–mesenchymal transition, and a pro-inflammatory tumor microenvironment. Importantly, the toxicity profiles of different aromatic amines are not uniform, with o-toluidine and 4-aminobiphenyl exhibiting particularly potent carcinogenicity.

Individual susceptibility is significantly modulated by genetic polymorphisms in metabolic enzymes, most notably the NAT2 slow acetylator phenotype, which synergizes with smoking to substantially elevate bladder cancer risk. Polymorphisms in NQO1, SULT1A1, and protective UGT1A variants further refine individual risk profiles. Risk prediction models integrating genetic and clinical markers show promise but require validation across diverse populations.

Despite substantial progress, several critical knowledge gaps persist. These include the health implications of low-dose, multi-amine combined exposures; the lack of a unified framework for comparing carcinogenic potency across different amines; inadequate confounder control in epidemiological studies. And the translational gap between experimental biomarkers and validated human biomonitoring applications. Future research should prioritize multi-omics integration to elucidate complete molecular networks and identify novel biomarkers, systematic investigation of low-dose combined exposures to inform realistic risk assessment, validation of polygenic risk scores for stratified screening of high-risk populations, development of non-invasive biomonitoring platforms for dynamic early warning, and evaluation of chemopreventive agents in targeted intervention trials. Ultimately, translating these mechanistic insights into an integrated prevention framework will be essential for reducing the disease burden of aromatic amine-related bladder cancer.
